# First catheter‐based high‐density endocardial 3D electroanatomical mapping of the right atrium in standing horses

**DOI:** 10.1111/evj.13265

**Published:** 2020-05-14

**Authors:** Eva Hesselkilde, Dominik Linz, Arnela Saljic, Helena Carstensen, Rayed Kutieleh, Thomas Jespersen, Prashanthan Sanders, Rikke Buhl

**Affiliations:** ^1^ Department of Biomedical Sciences Faculty of Health and Medical Sciences University of Copenhagen Copenhagen Denmark; ^2^ Centre for Heart Rhythm Disorders Royal Adelaide Hospital University of Adelaide Adelaide Australia; ^3^ Department of Veterinary Clinical Sciences Faculty of Health and Medical Sciences University of Copenhagen Copenhagen Denmark; ^4^ Abbott Medical Adelaide Australia

**Keywords:** horse, atrial arrhythmia, high‐density endocardial mapping, 3D electroanatomical mapping, HD Grid

## Abstract

**Background:**

Three‐dimensional electroanatomical mapping is of potential interest in equine cardiology to identify arrhythmia mechanisms, characterise electroanatomical substrates and guide ablation strategies.

**Objectives:**

To describe three‐dimensional electroanatomical mapping in standing horses.

**Study design:**

Research methodology, proof of concept study.

**Methods:**

Four Standardbred horses (2 geldings, 2 mares, median age 4.5 [4‐9] years, mean bodyweight 485 [440‐550] kg) were sedated and placed in stocks. Via the jugular vein, a high‐density multipolar grid catheter (Advisor^™^ HD Grid Mapping Catheter with EnSite VelocityTM, Abbott Medical) was used for endocardial mapping of the right atrium. The P‐wave on the surface ECG was used as a timing reference for simultaneous local activation time‐ and bipolar voltage‐mapping. For a positional reference a 10‐pole catheter (Abbott Medical) was placed in the caudal vena cava.

**Results:**

Endocardial right atrial mapping guided by the three‐dimensional mapping system and local electrograms was successfully performed in all four horses. A median of 32719 [25499‐65078] points, covering the entire right atrium, were collected. Three‐dimensional electroanatomical mapping provided detailed information about activation patterns and electrogram‐characteristics of the sinoatrial node, intervenous tubercle and cavotricuspid isthmus. Additionally, transvenous biopsy forceps connected to the mapping system were visualised on screen to guide biopsy collection.

**Main limitations:**

The feasibility of electroanatomical mapping for the left atrium and in larger breeds requires further study.

**Conclusions:**

High‐density three‐dimensional electroanatomical mapping of the right atrium is feasible in the standing horse.

## 1 INTRODUCTION

Atrial fibrillation (AF) is the most common pathological arrhythmia in both humans and horses.^1^, ^2^ In humans, percutaneous catheter‐based procedures are used to identify and characterise the underlying mechanisms of the arrhythmia and to guide targeted ablation interventions to treat the arrhythmia. To assist with these procedures, three‐dimensional (3D) electroanatomical mapping systems have been developed to create 3D models based on the anatomy of the individual heart. Local bipolar electrograms (EGMs) carrying local activation, bipolar and unipolar voltage (EGM amplitude) information are projected on the 3D model. The amplitude and morphology of EGMs can be used to characterise the extent of the underlying arrhythmogenic structural remodelling, including slowed conduction, low‐voltage areas, focal activation and re‐entry circuits. Peak‐to‐peak voltage (Vpp) of local bipolar EGMs (voltage mapping) are used to quantify areas of low voltage. In addition to voltage mapping, the activation and propagation of impulses (activation mapping) can help to better understand arrhythmia mechanisms by distinguishing between re‐entry mechanisms or local activation by ectopic foci.

For a further in‐depth characterisation of the myocardial tissue, the 3D mapping system also enables connection of biopsy forceps to the mapping system to visualise and guide collection of endocardial biopsies from areas of interest, for example low‐voltage areas in the 3D electroanatomical map.^3^

The use of 3D electroanatomical mapping in horses is sparsely described^4^, ^5^ but was recently used to assist in ablation of an anaesthetised horse with atrial tachycardia originating from the right atrium.^6^, ^7^ It remains unclear whether sophisticated 3D high‐density (HD) mapping techniques can be used in standing horses. This study aimed to test the feasibility of 3D HD endocardial electroanatomical mapping of the right atrium in standing horses. In addition, we aimed to evaluate conduction patterns during intrinsic atrial activation as well as low‐voltage areas using a 16 electrode HD electrode grid catheter. Furthermore, we tested whether biopsy forceps could be connected to the mapping system and followed on screen in order to guide transvenous endocardial biopsy collection.

## 2 METHODS

### 2.1 Study population

In this proof‐of‐concept study we included four Standardbred horses (2 geldings, 2 mares, median age 4.5 [4‐9] years, mean bodyweight 485 [440‐550] kg). All animals were considered healthy based on clinical and biochemical examinations and no abnormalities were found from electrocardiographic (ECG) or echocardiographic examination. The two mares were teaching horses stabled at the University Hospital, whereas the geldings were recently retired racehorses scheduled for euthanasia for noncardiac‐related reasons.

### 2.2 Preparation of the horses

**Figure 1:**
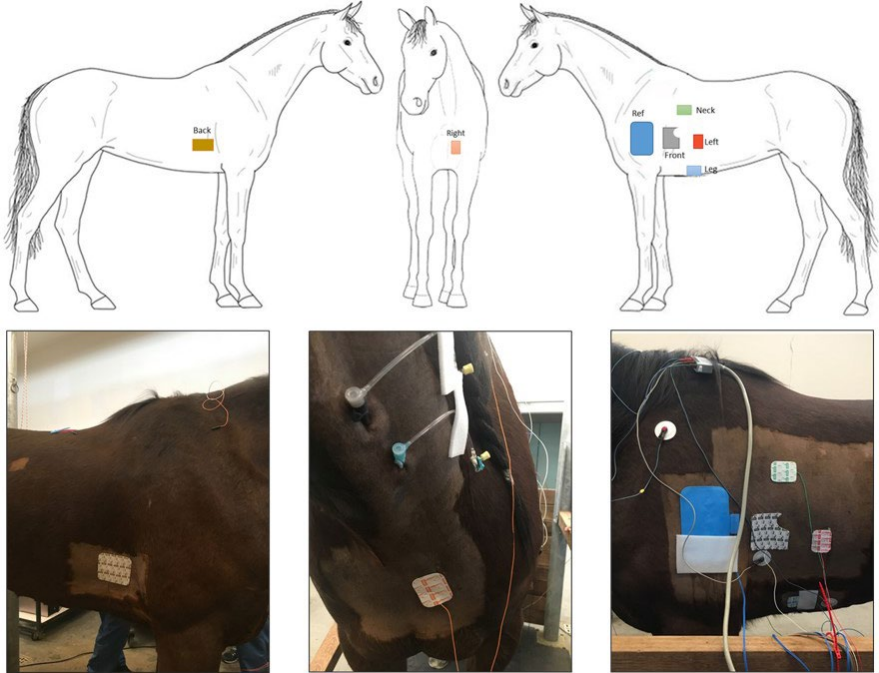
Placement of surface electrodes. The electrodes create an impedance field allowing intracardiac catheters to be located and visualised in the mapping system

Electrophysiological studies were performed in standing horses sedated with detomidine (10 µg/kg, Domosedan^®^), butorphanol (10 µg/kg, Torbugesic^®^) and a constant rate infusion of xylazine (1.0 mg/mL, Xysol^®^). An indwelling urinary catheter was placed to reduce movement of the horse during urination. Catheter based electroanatomical mapping of the right atrium (RA) was performed using a 3D electroanatomical cardiac mapping system; EnSite Velocity^™^ (Abbott Medical). The EnSite Velocity^™^ system uses surface electrodes to create an impedance field around the transthoracic area to locate and visualise intracardiac catheters. An intracardiac catheter was placed in a stable position for an anatomical intracardiac reference. The six surface electrodes and a system reference patch were placed orthogonally around the heart along the X (left‐right), Y (neck‐leg) and Z (front‐back) axis. As the heart of the horse is orientated in an up‐right position in the thorax with the right part of the heart rotated cranially, the surface electrodes could be placed on the horse while respecting the X, Y and Z axis of the heart. The adapted sticker location resulted in a change in projection so that the equine anterior‐posterior (AP) is comparable to a modified human right anterior oblique (RAO) view. This has to be considered when operating the 3D electroanatomical cardiac mapping system. The surface electrodes were placed as described in Table 1 and shown in Figure 1. The electrodes were placed on shaved skin and held in place with glue and adhesive foam. Two introducer sheaths (Radiofocus^®^ Introducer II, 8/10 F, 10 cm, Terumo) were placed in the left jugular vein to give access to the heart.

**Table 1:** Anatomical position of surface electrodes

Electrode	Anatomical position
Back	Right side of the thorax behind the shoulder
Front	Left side, behind the shoulder
Neck	6th intercostal space at level of the heart base
Leg	Caudal to the xiphoid process 10 cm left to the midline
Right	Left descending superficial pectoral muscle
Left	9th intercostal space, left side, level of the heart
Reference	Left triceps muscle

Echocardiography was initially used to guide the intracardiac reference catheter as well as the mapping catheter to the caudal vena cava. Right parasternal short axis view with dorsal probe angulation and variable rotation (+60° to +80°) from the fourth and third intercostal space enabled visualisation of caudal and cranial vena cava, intervenous tubercle, atrial septum and tricuspid valves as previously described.^8^ When the reference catheter was placed in the stable position and the mapping catheter was located in the right atrium, the right atrial electroanatomical mapping procedure was guided by the 3D electroanatomical mapping system.

### 2.3 Electroanatomical mapping protocol

**Figure 2:**
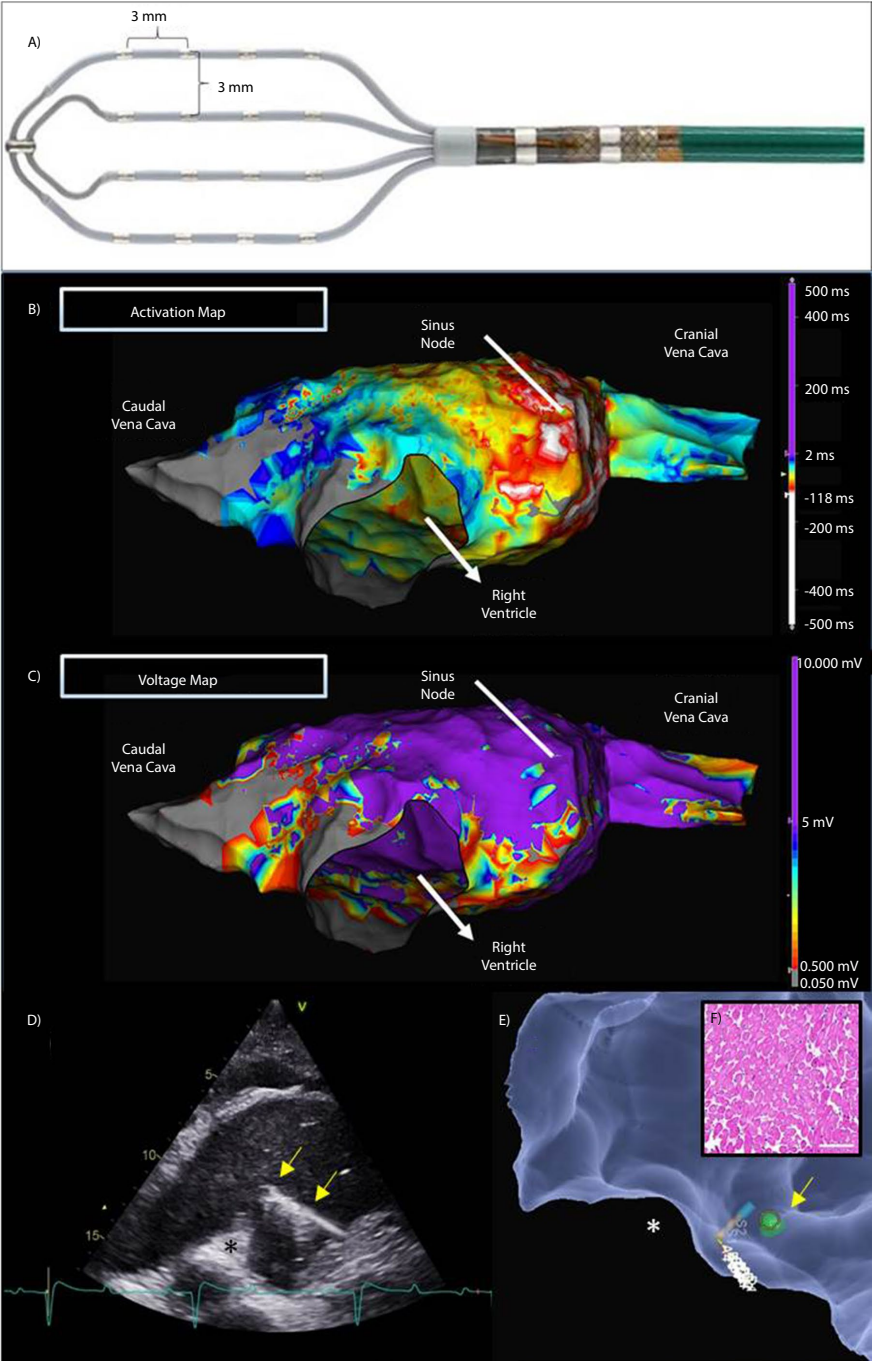
Advisor™ HD Grid Mapping Catheter (A, E). Representative activation (B) and voltage (C) map of the right atrium. Colour scale bar indicates activation time (ms) with the earliest activation in the sinus node (B) and voltage amplitudes (mV) of local electrograms (C). Biopsy forceps (yellow arrow) was used to collect tissue (F) from areas at interest. The biopsy forceps (yellow arrow) could be located both on echocardiography (D) and in the EnSite Velocity^™^ system (green mark, E). *Intervenous tubercle

The following catheters were utilised for mapping: (a) 6 F 10 pole catheter with 2‐5‐2 mm interelectrode spacing (Livewire^™^, Abbott Medical) positioned within the caudal vena cava for an intracardiac anatomical reference; (b) 16 electrode Advisor^™^ HD Grid Sensor Enabled Mapping Catheter (Abbott Medical), which has four 2.5 F splines configured in parallel with four electrodes on each spline (Figure 2, panel A). The electrodes are 1 mm in size with 3‐3‐3 mm equidistant inter‐electrode spacing. Local bipolar EGMs in combination with echocardiography was used to guide the catheters into the right atrium.

Automated HD 3D reconstructions of the RA were created using the Advisor^™^ HD Grid catheter. Simultaneously, RA activation and substrate voltage maps during sinus rhythm (including beats with second degree AV block) were created. The largest amplitude of a bipolar EGM is recorded when the bipolar electrodes are positioned parallel to the activation wavefront. The unique grid design of the Advisor^™^ HD Grid catheter and its use in conjunction with the HD wave algorithm allowed for EGM data to be recorded in orthogonal directions independent of catheter orientation. This allows the largest voltage for an orthogonal bipole pair to be included in the electroanatomical map.

Local activation times (LAT) and Vpp EGMs were acquired sequentially using the EnSite Velocity^™^ AutoMap^™^ software (Abbott Medical), which is a criteria‐based automated mapping software algorithm. The software utilises five main criteria to either accept or reject a mapping data point based on the following metrics; (a) Morphology match (60% to intrinsic sinus P‐wave), (b) Cycle length tolerance (OFF) (c) Speed limit (speed of catheter movement = 15 mm/s) (d) Distance Threshold (how far catheter has to move before collecting additional points = 0.05 mm) (e) Signal‐to‐Noise Ratio (= 4). Bipolar EGMs were bandpass filtered between 30 and 300 Hz and sampled at 203.45 Hz and their anatomical location annotated on the created 3D RA geometry map.

RA voltage thresholds were set between 0.05 and 0.5 mv where EGMs with a peak‐to‐peak voltage of less than 0.05 mV were classified as scar or electrically silent; between 0.05 and 0.5 mV as low‐voltage areas and >0.5 mV considered healthy tissue.^9^

To ensure that the complete maps only contained points collected while the HD Grid had proper contact to the endocardial wall the following projection settings were used: (a) Interior projection = 7 mm, (b) exterior projection = 7 mm.

The AutoColor^™^ (Abbott Medical) tool was used to visualise the right atrial LAT map. With this tool the colour white marked the point(s) collected with the earliest activation and purple was applied to the point(s) with the latest activation, with all colours on the colour bar (Figure 2B) applied to the points in between. This creates a colour map showing the direction of a sinus rhythm wavefront.

### 2.4 Biopsy and further procedures

After the electroanatomical map was created the HD Grid was removed and through an 8 F introducer sheath (Flexor^®^ check‐Flo^®^ Introducer Set, 90 cm, COOK Medical) biopsy forceps (Endomyocardial biopsy forcep, 2.4 mm × 105 cm, 7.5 F, Argon Medical) were inserted under echocardiographic guidance.^10^ A metal clip connected the forceps and the mapping system and the location of the biopsy forceps in the heart could be integrated in the 3D electroanatomical map. This allowed real‐time guidance of the forceps through the 3D anatomical map. Biopsies were paraffin‐embedded, sectioned and stained with H&E as previously described.^11^

The two retired racehorses were scheduled for euthanasia (140 mg/kg pentobarbital [Euthanasol^®^] IV) while the two teaching horses went back to stables to recover. Post‐operatively the horses were given 1.1 mg/kg flunixin (Finadyne^®^), and, to avoid intestinal impaction, the horses were treated with 1 L of paraffin oil and 5 L of electrolyte water through nasogastric intubation. No anti‐coagulant treatment was given.

## 3 RESULTS

Although designed for human use, the EnSite Velocity^™^ 3D electroanatomical cardiac mapping system in combination with the Advisor^™^ HD Grid catheter was feasible for electroanatomical mapping in standing sedated horses. The reference electrodes on the skin had good contact and the size of the horse did not prevent collection of data. A step‐wise approach was followed to create the RA electroanatomical map: (a) Guided by echocardiography and bipolar EGMs, the 10‐pole catheter was advanced into the RA while collecting data for the anatomical map. During mapping of the RA, the cranial and caudal vena cava were identified as structures cranial and caudal of the RA without EGMs. The azygos vein is a venous structure at the posterior side of the RA without EGMs. The right ventricle was identified when ventricular signals were recorded from the HD Grid. The tricuspid valve was identified by echocardiography and EGMs of the tricuspid valve anulus (atrial and ventricular signals). Cannulation of the CS ostium on the inferior aspect of the interatrial septum between the caudal vena cava and the tricuspid valve was attempted. Anatomical structures were confirmed by echocardiography and were used to guide orientation of the map. (b) The HD Grid was then introduced into the RA to create an electroanatomical map, simultaneously collecting both LAT and Vpp maps.

In the first horse, we aimed to cannulate the coronary sinus (CS) with the 10‐pole catheter to obtain a stable intracardiac reference to reduce the effect of location impedance shifts and drifts during the procedure. However, engaging the CS with the 10‐pole catheter proved not possible due to several valves located at the ostium of the CS (confirmed post‐mortem, data not shown). As a result, the system reference patch was used as a reference for intracardiac catheter location. This method worked well for the first horse as an accurate electroanatomical map was created and remained stable throughout the procedure. However, in the second horse, catheter location shifts were observed when creating the electroanatomical maps which was identified by the collection of two identical but parallel geometries. To correct for these shifts, echocardiographic and EGM guidance was used to place both catheters in two known locations in the RA such as the cranial and caudal vena cava and a manual alignment of the catheters to the correct 3D geometry was performed. The secondary RA geometry was subsequently deleted. Based on the learnings from horse 2, the 10‐pole catheter was placed in the caudal vena cava as stable intracardiac reference in the following horses. The 3D mapping system can shadow the intracardiac reference catheter which helps to detect shifts of the 3D anatomical maps and allow simple alignment of the maps. Median mapping time was 85 [55‐118] minutes. A median of 32719 [25499‐65078] EGMs were collected in each animal of which a median of 3048 [2731‐5546] were used and analysed (Table 2). Three‐dimensional anatomical maps were created where the right atria were bordered by anatomical structures (the cranial and caudal vena cava and the tricuspid valve) identified by the relative location to each other and by the interpretation of local EGMs. In addition to the geometric map, we were able to create activation maps showing propagation of the atrial impulses originating from the sinoatrial node (SAN) and the resulting activation wave front propagating through the atrium. Low‐voltage areas were usually found around the cavotricuspid isthmus, around the two caval veins and on the intra‐atrial septum. Figure 2 illustrates a representative activation (B) and voltage (C) map.

**Table 2:** Mapping data for each for the horses included in the study. Included points were defined to be located within 7 mm of geometry surface. Low voltage was defined as <0.5 mV

	Case #1	Case #2	Case #3	Case #4
Horse characteristics				
Age	4	5	3	9
Weight	440	450	500	550
Sex	Gelding	Gelding	Mare	Mare
Mapping data				
Collected points	65 078	36 254	29 184	25 499
Included points	5546	1909	2731	3365
Areas of low voltage	Cranial caval vein, caudal caval vein, azygos vein, interatrial septum	Cranial caval vein, caudal caval vein, azygos vein, interatrial septum	Cranial caval vein, caudal caval vein, azygos vein, interatrial septum, lateral RA wall anterior to the azygos vein and crista terminalis as well as in between the azygos vein and caudal caval vein	Cranial caval vein, caudal caval vein, azygos vein, interatrial septum
Total mapping time (min)	118	83	87	55

Abbreviation: RA, right atrium.

The HD Grid and the biopsy forceps could be visualised on echocardiography and on the mapping system which facilitated mapping‐guided biopsy collection (Figure 2, panel D,E). H&E staining of the biopsies collected from the RA revealed that the biopsies contained myocardial tissue (Figure 2, panel F).

## 4 DISCUSSION

In this proof‐of‐concept study, we assessed the feasibility of mapping the RA of four standing horses using a 3D electroanatomical cardiac mapping system developed for standard use in humans. The main findings of this study are as follows: (a) It is possible to create 3D geometric maps of the RA in standing horses; (b) The HD Grid catheter can create detailed activation and voltage maps of the RA; (c) Mapping‐guided biopsy collection is feasible in standing horses; (d) All horses tolerated the procedure well and the two horses intended for survival recovered with no side effects.

Simple catheter‐based atrial electrophysiological procedures, as well as catheter‐based transvenous electrical cardioversion of AF, are well described in horses.^12^, ^13^, ^14^, ^15^, ^16^ In contrast to humans and small animals, where fluoroscopy is often used to navigate catheters, catheter placement is generally guided by electrical signals and echocardiography in horses, as the width of the thorax makes chest fluoroscopy challenging.^8^ In addition, chest fluoroscopy often requires general anaesthesia, while many electrophysiological procedures can be performed in standing horses.

Recently, Van Loon and co‐workers described how 3D electroanatomical mapping was used to guide ablation of a right‐sided atrial tachycardia in an anaesthetised horse.^6^ Activation mapping of right atrial arrhythmias may help to identify arrhythmia mechanisms. In humans, typical atrial flutter is due to re‐entry that is dependent on an area of slow conduction through the cavotricuspid isthmus^17^ whereas atrial tachycardias often originates from anatomical structures with ectopic activities.^18^ The identification of the underlying arrhythmia mechanism is important to guide ablation and treat the arrhythmia.

However, 3D electroanatomical mapping has not yet been described in standing horses, where electrophysiological measurements can be recorded without the periprocedural risks and additional financial costs that comes with general anaesthesia. We report herein on the first electroanatomical mapping procedure in standing sedated horses. Compared to other mapping systems, which require a magnet placed below the body, the EnSite Velocity^™^ system used in this study uses patches placed on the body surface, to create an impedance field to locate and visualise intracardiac catheters within the heart. Based on clinical experiences in humans, a comparable mapping procedure in standing horses using a magnetic‐based location system would most likely not be possible given constant movement artefact.

Further studies are needed to determine prevalence and mechanisms of common arrhythmias in horses and to test, whether high‐density electroanatomical maps (a median of 32719 [25499‐65078] points covering the entire right atrium) as performed in this proof‐of‐concept study are sufficient to allow targeted ablation procedures in horses. Theoretically, mapping of the left atrium should also be possible by trans‐septal or retrograde approaches. Particularly in the case of AF, 3D electroanatomical mapping of the pulmonary veins would be interesting to guide pulmonary vein isolation procedures.

Transvenous endomyocardial biopsies are described both in human cardiology and in veterinary medicine as a diagnostic approach for myocarditis, cardiomyopathy, cardiac tumours or toxic cardiomyopathy.^13^ In this study, we showed that it is possible to connect a biopsy forceps to the mapping system and collect endocardial biopsies from areas at interest. Future studies can use this method to identify low‐voltage areas and through histology perform targeted analysis of the affected tissue. In addition to clinical applications, electroanatomical mapping may also have applications in research projects. Electroanatomical mapping and mapping guided biopsy collection may support characterisation of structural arrhythmogenic substrates in future studies.

### 4.1 Limitations

Despite the difference in size and cardiac anatomy between humans and horses, it was possible to create 3D anatomical maps with the shape of an equine right atrium and follow the activation and propagation of the electrical impulses. It is however important to choose adequate settings and solely include points in close proximity to the surface. In this study this led to the exclusion of many collected points and critical evaluation of the maps are necessary. Larger mapping catheters with a wider curve of the steerable mechanism would be required to reduce the points without wall contact. In general, the procedure of 3D electroanatomical mapping is complex and requires specialised equipment and personal. The horses used in this study were Standardbred horses with an average weight of 485 kg. In these horses, size was not an issue and all catheters and cables were compatible, and no custom‐made equipment were needed. However, it remains unknown whether this technique can be used in larger breeds. Another limitation of this study is the low number of animals included, which cannot facilitate accurate estimation of complications and safety issues.

### 4.2 Conclusions

In summary, 3D electroanatomical mapping of the right atrium is feasible in the standing horse and may be helpful in characterising the underlying mechanisms for right atrial arrhythmias as well as guiding ablation strategies in the future.

## CONFLICT OF INTERESTS

Rayed Kutieleh is employed by Abbott Medical.

## AUTHOR CONTRIBUTIONS

E. Hesselkilde, D. Linz and R. Kutieleh contributed to study design, study execution, data analysis and interpretation, and preparation of the manuscript. A. Saljic contributed to study execution, data analysis and interpretation, and preparation of the manuscript. H. Carstensen, T. Jespersen, P. Sanders and R. Buhl contributed to study design and study execution. All authors gave their final approval of the manuscript.

## ETHICAL ANIMAL RESEARCH

Local ethic committee and The Danish Animal Experiments Inspectorate approved the study (license number: 2016‐15‐0201‐01128).

## OWNER INFORMED CONSENT

Written owner‐informed consent was obtained prior to the study.

## Data Availability

The data are available from the corresponding author on reasonable request.
