# Supplementary material for: First catheter‐based high‐density endocardial 3D electroanatomical mapping of the right atrium in standing horses
Source: Equine Vet J. 2020 May 14;53(1):186–93. doi: 10.1111/evj.13265 (PMC7818172; doi:10.1111/evj.13265)
Supplement: Supplementary file 1 [file EVJ-53-186-s001.docx]

**EVJ-TN-19-292.R2**

**首例经导管的直立马匹右心房心内膜高密度电解剖绘图**

Eva Hesselkilde^1*^, Dominik Linz^2*^, Arnela Saljic^1^, Helena Carstensen^3^, Rayed Kutieleh^4^, Thomas Jespersen^1^, Prashanthan Sanders^2#^ and Rikke Buhl^3#^.

^1^生物医学科学系，健康与医学科学院， 哥本哈根大学, 丹麦; ^2^心率异常研究中心，阿德莱德皇家医院和阿德莱德大学，阿德莱德，澳大利亚; ^3^临床兽医科学系，健康与医学科学院，哥本哈根大学, 丹麦 ^4^雅培医疗，澳大利亚.

**关键词：**马；心房心律失常；高密度心内膜造影术；3D电解剖绘图；高清网格

**栏外标题：**直立马匹心内膜3D电解剖绘图

*通讯作者 email: rib@sund.ku.dk

*Eva Hesselkilde 和 Dominik Linz 为共同第一作者

^#^ Prashanthan Sanders 和Rikke Buhl 为共同最后作者

**总结**

**背景：**三维电解剖绘图在马心脏学中具有很多潜在意义，能帮助识别心律失常的机制，确定特异性电解剖基质并指导消融策略。

**目的：**描述直立马的三维电解剖绘图

**研究设计：**概念验证研究。

**方法：**对4匹标准马（2匹骟马，2匹母马，年龄中位数4.5[4-9]岁，平均体重485[440-550]公斤），放入保定架，进行镇静处理。通过颈静脉，使用高密度多极网格导管（Advisor™HD网格映射导管，EnSite VelocityTM, 雅培医疗）进行右心房的心内膜定位绘图。将体表心电图上的p波作为同步局部激活双极电压绘图的时间参考。在尾腔静脉放置一个10极导管(雅培医疗)，用于定位参考。

**结果：**所有4匹马均在三维定位绘图系统和局部心电图的指导下成功地完成了心内膜右房造影。收集到中位数32719[25499-65078]个点，覆盖整个右心房。三维电解剖绘图提供了窦房结、室间结节和三尖瓣峡部的激活模式和电图特征的详细信息。此外，活检钳可以经静脉，连接到绘图系统并显示在屏幕上，以指导活检收集。

**主要局限性：**左心房及大动物的电解剖绘图的可行性有待进一步研究。

**结论：**直立马右心房高密度三维电解剖绘图是可行的。
